# Workplace discrimination and its relationship with organizational commitment among the surgical technologist: A national cross-sectional study in Iran

**DOI:** 10.3389/fpsyg.2022.1047153

**Published:** 2023-01-11

**Authors:** Esmaeil Teymoori, Negar Shahkarami, Maryam Ghanavati, Zahra Maleki, Armin Fereidouni

**Affiliations:** ^1^Department of Operating Room Technology, Faculty of Paramedical Sciences, AJA University of Medical Sciences, Tehran, Iran; ^2^School of Allied Medical Sciences, Fasa University of Medical Sciences, Fasa, Iran; ^3^Department of Operating Room Technology, Faculty of Nursing and Midwifery, Ahvaz Jundishapour University of Medical Sciences, Ahvaz, Iran; ^4^Student Research Committee, Shiraz University of Medical Sciences, Shiraz, Iran; ^5^Trauma Research Center, Shahid Rajaee (Emtiaz) Trauma Hospital, Department of Operating Room Technology, Faculty of Nursing and Midwifery, Shiraz University of Medical Sciences, Shiraz, Iran

**Keywords:** workplace discrimination, organizational commitment, operating room, surgical technologist, nurse

## Abstract

**Background:**

Discrimination in healthcare centers can reduce care quality and job satisfaction, weaken the morale of the personnel and increase the cost of health care and finally lead to turnover intention. Discrimination in hospitals can affect the work outcomes and performance indicators of nurses such as their organizational commitment. Due to the vital role of surgical technologists in surgery and justice as the basis of effective performance, as well as the role of organizational commitment in care quality, the present study was conducted aimed to determine the level of workplace discrimination from viewpoint of the surgical technologists and its relationship with the organizational commitment.

**Methods:**

This cross-sectional study was conducted on 615 surgical technologists in 8 metropolises of Iran in 2022. The sampling method in this study was multi-stage. The data collection tools included three questionnaires (demographic information, workplace discrimination, and Allen and Meyer’s organizational commitment). Data were collected within 2 months and analyzed by SPSS version 22. Descriptive and inferential analyzes including independent *t*-test and analysis of variance were used for data analysis. *p*-value ≥ 0.05 was considered significant.

**Results:**

The average age of the participants in the study were 29.90 ± 5.83 years with the participation of 464 women. The results of the data analysis showed an inverse and significant correlation between workplace discrimination and organizational commitment (*r* = −0.149, *p*-value < 0.001). The mean and standard deviation for workplace discrimination was 108.20 ± 11.53, which is average. Also, the mean and standard deviation for total organizational commitment was 100.56 ± 19.14, which is higher than the average.

**Conclusion:**

According to the results of this study, managers need to pay attention to reduce discrimination in the operating room and establish organizational justice, to improve the motivation of surgical technologists and the quality of their performance. It is also suggested that operating room managers and supervisors, by creating a safe, confidential, and fearless environment to encourage the personnel to express what they understand about discriminatory conditions.

## 1. Introduction

The word “discrimination” literally means “unfair behavior with a person or a group of people different from others” and “ignoring equal opportunities such as education, employment, housing, and health care” (Home, 2019). Workplace discrimination is one of the factors affecting the mental health and job performance of human resources in an organization (Chou and Choi, 2011). According to Hahn and Wilkins, workplace discrimination occurs when a group of personnel in the organization are less considered by others (Hahn and Wilkins, 2013). Discriminatory behaviors usually result from factors related to gender, race, religion, social status, or political views (Kingma, 1999; Arumugham, 2017). A study by Ogden et al. showed that the majority of health care workers considered racism and gender as the main causes of discrimination in hospitals (Ogden et al., 2005).

Hospitals as health care organizations are among the environments where discrimination is high (Coombs and King, 2005). Perceived discrimination in medical centers can reduce the quality of care, increase the cost of health care (Halley et al., 2018), reduce job satisfaction, weaken the morale of the personnel (Newman, 2014), and finally may lead to turnover intention (Klinner and Walsh, 2013). Nunez-Smith et al., by conducting a study on physicians in the United States, found that workplace discrimination was the main cause of dissatisfaction, leaving work (Nunez-Smith et al., 2009).

In Iran, surgical technologists are members of the surgical team and work as circulating and scrub nurses in the operating room (Imani et al., 2022). The results of studies show that nurses consider workplace discrimination as one of the main barriers to inter-professional relations and inequality in authority and disrespect for their position as factors of discrimination (Osuji et al., 2014; Baptiste, 2015; Valizadeh et al., 2015). In addition, one of the most serious and common problems of nurses is discrimination between them and physicians (ZareKhafri et al., 2022), which leads to inappropriate behaviors on the part of nurses and physicians. This type of behavior acts as a barrier to cooperation (Hosseinzadegan et al., 2021). In Iran, the position of physicians is much higher than that of nurses, and nurses have lower salaries, and the lack of promotion opportunities increases the gap between these two professions (Zamanzadeh et al., 2013). Discrimination in hospitals can affect the work outcomes and performance indicators of nurses such as their organizational commitment (Dinmohammadi et al., 2010; Slany et al., 2014; Rhead et al., 2021).

Organizational commitment is an effort that all personnel from the lowest to the highest rank make in the way of obtaining the organization’s goals (Hannani et al., 2020). Persons with high organizational commitment prefer to stay in their jobs even in difficult situations. Since in the organization, personnel are considered as a key asset, so their organizational commitment is highly regarded (Salehi and Dadgar, 2016). The implementation of optimal health services depends on the health care worker’s performance. To achieve this goal, it is not enough to have permanent personnel in the organization and human resources with high organizational commitment are needed (Rahmati and Seyfi, 2021). Specialist and committed personnel are one of the vital needs of any organization, whose presence, in addition to improving the quality of work life (Chanvibol et al., 2020) and Job Satisfaction (Sangperm, 2017; Moonsri, 2018), can reduce absenteeism and delay, increase the performance of the organization, and finally achieve the goals of the organization (Teymoori et al., 2022). In this regard, Hannani et al. reported the level of organizational commitment in operating room personnel as average. In this study, most of the people remained in their jobs due to their high costs of leaving the organization (Hannani et al., 2020).

Surgery is a key part of any health system (Danson et al., 2020). As members of the surgical team, surgical technologists play an effective role in patient admission, surgical intervention, and patient care in the operating room, and their performance guarantees the patient’s safety and the quality of his care (Imani et al., 2022). For the professional and scientific role of surgical technologists in surgery, the importance of justice as the basis of effective performance, and the role of organizational commitment in the quality of patient care, it is important to identify discrimination in the operating room and its relationship with organizational commitment. Therefore, the present study was conducted aimed to determine the level of workplace discrimination from viewpoint of surgical technologists and its relationship with organizational commitment.

## 2. Materials and methods

### 2.1. Study design and participants

This cross-sectional study was conducted on 615 surgical technologists from October 21, 2021, to June 19, 2022, in 8 metropolises in Iran. The sample size is based on the study results of ZareKhafri et al. (2022) with a standard deviation of 6.7 using the formula $n\geq {\left[\frac{z^{1}-\frac{\alpha^{\sigma }}{2}}{d}\right]}^{2}$ at confidence level of 95% and error margin of 0.57.

**Figure 1:**
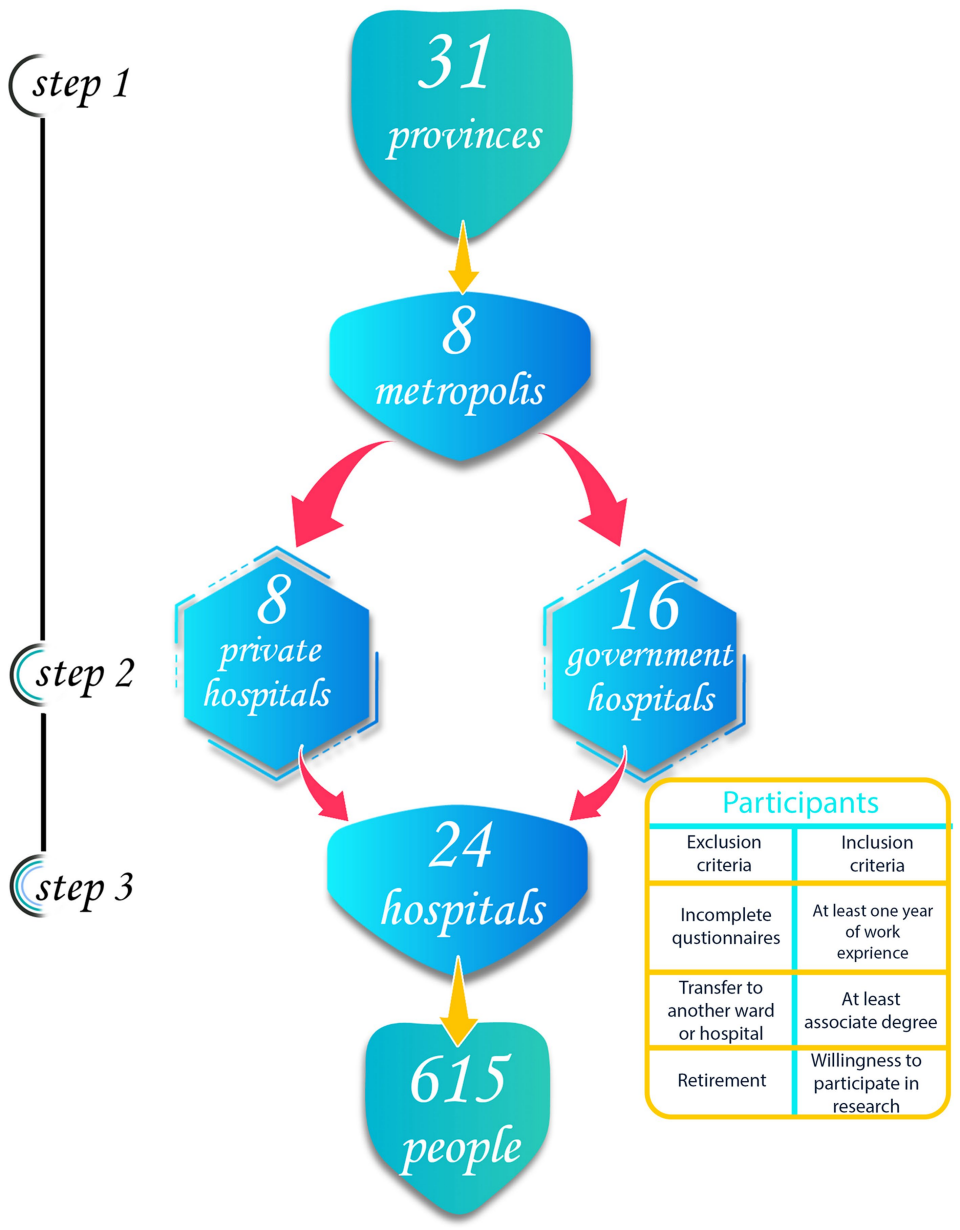
Multi-stage sampling method.

The sampling method in this study was multistage (Figure 1). First, 8 metropolises were selected among 31 provinces by simple random method (using lottery). Next, based on the type of hospital (private/public hospital), 2 hospitals (1 public hospital and 1 private hospital) were selected. Then, in these hospitals, based on the number of surgical technologists in the operating room, people were selected in a stratified manner through identification numbers. The link of the online questionnaires was sent to them through social networks (WhatsApp and Telegram). Follow-up of people to answer was done up to 3 times, and if they did not want to cooperate in the study, the next random number was selected. Inclusion criteria included at least an associate’s degree in surgical technology and more than 1 year of work experience in the operating room and willingness to participate in the study. Exclusion criteria were incompleted the questionnaires, and transfer to another hospital or retirement.

For designing the online questionnaires, IP filtering was used to prevent repeated answers, and the participants could refuse to complete the questionnaires at any time. After entering the questionnaires link and before completing the questionnaires, the participants were informed about the objectives of the study, and a written informed consent form was obtained electronically before completing questionnaires.

### 2.2. Measuring instrument

In the present study, there are 3 questionnaires including a demographic information questionnaire (age, gender, marital status, level of education, employment status, type of hospital, and work experience), a workplace discrimination questionnaire (ZareKhafri et al., 2022), and Allen and Meyer’s organizational commitment questionnaire (Teymoori et al., 2022) were used.

#### 2.2.1. Workplace discrimination questionnaire

This questionnaire has 33 questions and 5 dimensions, which were scored on a Likert scale from completely disagree (score 1) to completely agree (score 5). Items 4, 14, 18, and 19 were scored in reverse. The total scores of the questionnaire range from 33 to 165, and higher scores indicate more workplace discrimination. Factor 1 as horizontal and vertical discrimination had 14 items (6, 14 to20, 22 to 27), Factor 2 as consequences of discrimination had 6 items (28, 29, 30, 31, 32, and 33), Factor 3 as inequality due to differences in employment, education, ethnicity had 4 items (8, 9, 10 and 21), Factor 4 as unequal job promotion had 4 items (4, 5, 12 and 13) and Factor 5 as gender and culture discrimination had 5 items (1, 2, 3, 7, and 11). The validity of the questionnaire has been confirmed using face validity (quantitative and qualitative), content validity (quantitative and qualitative), and construct validity (factor analysis). The interval of 2 weeks was 0.761 (p = 0.01) and the internal consistency of the questionnaire was α = 0.813 (ZareKhafri et al., 2022).

#### 2.2.2. Organizational commitment questionnaire

The current questionnaire has 24 questions that were designed in 1991 (Victor and Cullen, 1988). This questionnaire evaluates the components of emotional commitment (8 questions), continuous commitment (8 questions), and normative commitment (8 questions). This questionnaire is based on a seven-point Likert scale including (completely disagree, somewhat disagree, slightly disagree, have no opinion, slightly agree, somewhat agree, and completely agree) with scores from 1 to 7, respectively. Questions 4,5, 6, 8, 9, 18, 19, 21, and 24 are scored in reverse. In this way, for the option I completely agree, the score is 1, and in the same way, for the option, I completely disagree, which is considered a score of 7. The score range was from the lowest score of 24 to the highest score of 168. The higher the score obtained from the total score of all statements, the higher the organizational commitment. The psychometrics of the Persian version of the Organizational Commitment Questionnaire has been confirmed in a study by Buyukzadeh et al. in Iran with Cronbach’s alpha of 0.84 (Boyokzadeh et al., 2017; Teymoori et al., 2022).

### 2.3. Data analyses

For data analysis, descriptive analyzes including frequency, percentage, mean, standard deviation, and analytical statistics were used for qualitative and quantitative data. Differences between subgroups were compared using the Chi-square test. The relationship between variables was investigated using Pearson’s correlation coefficient, independent t-test, and ANOVA test. The normality of all quantitative variables was investigated using the Kolmogorov–Smirnov test. The collected data were analyzed by SPSS version 22. *p*-value ≥ 0.05 was considered statistically significant.

### 2.4. Ethical considerations

The Ethics Committee of Shiraz University of Medical Sciences has approved the permission to conduct this study with the code of ethics IR.SUMS.NUMIMG.REC.1400.074 and access link https://b2n.ir/d15193. There is no need to mention the name of the participants in this study and the participants were explained the confidentiality of the information. The current study was designed based on the STROBE guidelines for observational studies.

## 3. Result

The average age of 615 participants was 29.90 ± 5.83 years. The majority were 464 women (75.4%) and 320 (52%) participants were married. An inverse and significant correlation was between age and workplace discrimination (*p*-value = 0.002, *r* = −0.12). In addition, an inverse and significant correlation was between work experience and workplace discrimination (*p*-value = 0.002, *r* = −0.12). A significant relationship was between marital status (*p*-value = 0.003), degree (*p-*value = 0.028), employment status (*p*-value = 0.003) with workplace discrimination. Also, an inverse and significant correlation was between work experience and organizational commitment (*p*-value = 0.002, *r* = −0.12). A significant relationship was between marital status (*p*-value = 0.003), type of hospital (*p*-value < 0.001), employment status (*p*-value < 0.001) with organizational commitment. Table 1 shows the details of demographic information and their relationship with workplace discrimination and organizational commitment.

**Table 1:** The relationship between the demographic information of the participants with the average workplace discrimination and organizational commitment (*n* = 615).

variables	Category	*n* (%)	workplace discrimination (Mean ± SD)	*p*-value	Organizational commitment (Mean ± SD)	*p*-value
Gender	Female	464 (75.4%)	108.61 ± 10.940	0.160	101.30 ± 98.26	0.090
	Male	151 (24.6%)	106.94 ± 13.121		98.26 ± 19.15	
Marital status	Single	295 (48%)	109.66 ± 11.47	0.003	98.20 ± 19.16	0.003
	Married	320 (52%)	106.85 ± 11.42		102.73 ± 18.88	
Educational degree	Associate degree	50 (8.1)	104.22 ± 11.90	0.028	105.66 ± 14.00	0.141
	Bachelor’s	509 (82.8)	108.68 ± 11.37		100.17 ± 19.44	
	Master’s	56 (9.1)	107.37 ± 12.83		99.53 ± 19.92	
Type of hospital	Educational	496 (80.7)	107.89 ± 11.88	0.128	102.32 ± 18.23	<0.001
	Private	119 (19.3)	109.49 ± 9.84		93.20 ± 21.07	
Work history (years)	<3	251 (40.8)	109.11 ± 11.55	0.180	99.99 ± 19.76	0.191
	3–7	162 (26.3)	108.16 ± 12.06		99.02 ± 19.72	
	>7	202 (32.8)	107.10 ± 10.99		102.49 ± 17.75	
Employment Status	Permanent	266 (43.3)	106.60 ± 11.87	0.003	104.00 ± 17.18	<0.001
	Contractual	349(56.7)	109.42 ± 11.11		97.93 ± 20.13	

According to the study results, the mean and standard deviation for total workplace discrimination was 108.20 ± 11.53, which is average. Also, the mean and standard deviation of total organizational commitment was 100.56 ± 19.14, which is higher than the average. The mean and standard deviation of dimensions of workplace discrimination and organizational commitment of surgical technologists are shown in Table 2.

**Table 2:** Dimensions of workplace discrimination and organizational commitment.

Variable	Dimension	Mean ± SD	Lower score	Highest score
Workplace discrimination	Vertical and horizontal discrimination	49.17 ± 5.32	48.75	49.59
	Consequences of discrimination	19.79 ± 5.01	19.39	20.18
	Organizational-cultural discrimination	12.13 ± 2.17	11.96	12.30
	Unfair promotion	14.08 ± 2.85	13.85	14.30
	Gender discrimination	13.94 ± 3.28	13.68	14.20
	Total workplace discrimination	108.20 ± 11.53	107.29	109.12
Organizational commitment	Affective commitment	32.78 ± 9.58	32.02	33.54
	Continuous commitment	38.53 ± 9.30	37.80	39.27
	Normative commitment	29.24 ± 7.20	28.67	29.81
	Total organizational commitment	100.56 ± 19.14	99.05	102.07

The results of the data analysis showed an inverse and significant relationship between workplace discrimination and organizational commitment (*r* = −0.149, *p*-value < 0.001).

In the present study, there is a significant correlation between dimensions of Vertical and horizontal discrimination (*p* < 0.001, *r* = −0.195), Unfair promotion (p < 0.001, *r* = −0.185) with Total Organizational Commitment. also, there is significant Correlation between dimensions Affective commitment (*p* < 0.001, *r* = −0.186) and Normative commitment (*p* = 0.001, *r* = −0.132) with Total workplace discrimination. The correlation between the dimensions of workplace discrimination and organizational commitment are shown in Table 3.

**Table 3:** Correlation between the dimensions of workplace discrimination and the level of organizational commitment of the operating room personnel.

Organizational commitment	Workplace discrimination					
	Vertical and horizontal discrimination	Consequences of discrimination	Organizational-cultural discrimination	Unfair promotion	Gender discrimination	Total workplace discrimination
Affective commitment	−0.216 <0.001	0.28 0.484	−0.010 0.805	−0.216 <0.001	−0.117 0.004	−0.186 <0.001
Continuous commitment	−0.061 0.134	0.017 0.666	0.014 0.735	−0.008 0.843	0.036 0.373	−0.013 0.752
Normative commitment	−0.153 <0.001	0.036 0.374	−0.012 0.765	−0.195 <0.001	−0.062 0.123	−0.132 0.001
Total Organizational Commitment	−0.195 <0.001	0.036 0.371	- 0.003 0.943	0.185- <0.001	−0.065 0.109	- 0.149 <0.001

In each table cell, the first-row number indicates the correlation coefficient (r) and the second-row number indicates the significance level (*p*-value).

## 4. Discussion

This study was conducted aimed to determine workplace discrimination from viewpoint of the surgical technologists and its relationship with organizational commitment. In the present study, the average of the vertical and horizontal discrimination dimensions has the highest score among the dimensions of discrimination in the operating room. Vertical discrimination is a type of discrimination that managers, for example, head nurses apply to their nurses, and horizontal discrimination refers to discriminatory behaviors between colleagues, for example, nurses in the same ward (ZareKhafri et al., 2022). Since this behavior is not due to superior job skills, it affects the care performance of operating room personnel (Shohani, 2019). The results of the present study are consistent with studies conducted by ZareKhafri et al. in Iran (ZareKhafri et al., 2022) and Ogden et al. in America (Ogden et al., 2005). In this regard, the results of qualitative studies in Iran have shown that discrimination exists as a concern among nurses for inter-professional cooperation (Valizadeh et al., 2015; Teymoori et al., 2022). In addition, discrimination unconsciously leads to aggressive behaviors that are destructive to the goals of the organization. If supervisors and managers in the health field are not aware of the importance of justice between nurses and operating room personnel, it is concluded that they do not work according to professional principles, which is against the goals of hospitals (Holley et al., 2019; Shohani, 2019).

In the present study, the mean organizational commitment among surgical technologists was found to be higher than average, which is consistent with studies by Teymoori et al. (2022) and Khachian et al. (2016), in Iran and Seren Intepeler et al. in Turkey (Seren Intepeler et al., 2019); and inconsistent with a study by Ahmad and Oranye on nurses (Ahmad and Oranye, 2010), which may be due to the different climate of the operating room compared to other wards.

According to the results of the present study, an inverse and significant relationship was between discrimination in the workplace and organizational commitment. This study showed that discrimination can affect organizational commitment. The existence of workplace discrimination causes a negative impact on duties and values, so that a person may be committed to the organization only to the extent of meeting their needs, as Hannani et al. observed in their study that due to the high cost of leaving the job and the rewards for staying at work, they stayed in the organization (Hannani et al., 2020). If high commitment prevails in the organization, personnel are willing to cooperate beyond the requirements of the existing organization (Meyer and Parfyonova, 2010). This means that persons will have a much higher sense of responsibility towards their duties (Shohani, 2019).

In this study, no significant relationship was found between gender discrimination and total organizational commitment. The gender discrimination among the participants was low on average. The study results were consistent with the study results of ZareKhafri et al. (2022). but inconsistent with studies by Coombs and King (2005) and Newman (2014). It seems that gender discrimination is low in Iran. Since in general gender discrimination can cause hatred of one gender and lead to inequality in rights and managerial positions between genders (ZareKhafri et al., 2022), and It also leads to a decrease in job satisfaction and employee performance (Kerdpitak and Jermsittiparsert, 2020), so ZareKhafri et al. in their study emphasizes the need to improve this organizational culture (ZareKhafri et al., 2022).

The study results showed a significant relationship between degree and workplace discrimination from viewpoint of the surgical technologists, which is inconsistent with a study conducted in Serbia. A study by Milutinović et al. showed that nurses with higher education are less affected by discriminatory factors than nurses with lower education (Milutinović et al., 2012). In addition, the results of a study in Iran showed that the degree cannot affect understanding the factors of discrimination (ZareKhafri et al., 2022). It seems that the differences may be due to the difference in the sample size with different degrees.

In the present study, a significant relationship was between employment status and workplace discrimination, so that people who are officially employed have a greater understanding of workplace discrimination, which is consistent with the previous study (ZareKhafri et al., 2022). In general, it seems that the level of expectations for the implementation of justice and elimination of discrimination in this group of personnel is higher than those who are contractually employed. Also, the study results showed that by increasing age and work experience, less discrimination is perceived by surgical technologists, which is consistent with a study by Nunez-Smith et al. (Nunez-Smith et al., 2009). This may be related to balancing their expectations by increasing age and work experience.

This study had some limitations. No similar study was found that investigated workplace discrimination in surgical technologists, so we attempted to use the related studies on the subject. Another limitation of this study was the self-report method of completing the questionnaire by the participants, which the researcher attempted to reduce by providing the necessary explanations of the study objectives. Similar studies have focused more on discrimination against nurses since the special setting of the operating room is completely different from the wards and the clinical care of nurses in terms of job description is not comparable to that of surgical technologists, this study specifically focuses on surgical technologists. Another strength of this study is the large sample size in a wide geographical region.

## 5. Conclusion

The study results showed discrimination as one of the main concerns of surgical technologists in inter-professional relationships, which can lead to a reduction in organizational commitment. For a correct understanding and management of the dimensions of discrimination and organizational commitment in the operating room, it is necessary for surgical technologists and relevant managers to be aware of the psychological and professional consequences of workplace discrimination. In addition, learning clinical skills during surgery is costly and time-consuming and if people leave their jobs after acquiring skills due to low organizational commitment, it will cost the organization a lot. Researchers suggested that, by providing a safe, confidential, and fearless environment, the managers of the medical centers should encourage the personnel to express what they understand about the discriminatory conditions and create the necessary motivations for the commitment of the personnel to the organization.

## Data availability statement

The raw data supporting the conclusions of this article will be made available by the authors, without undue reservation.

## Ethics statement

The Ethics Committee of Shiraz University of Medical Sciences has approved the permission to conduct this study with the code of ethics IR.SUMS.NUMIMG.REC.1400.074 and access link https://b2n.ir/d15193. There is no need to mention the name of the participants in this study and the participants were explained the confidentiality of the information. The current study was designed based on the STROBE guidelines for observational studies.

## Author contributions

AF and ET: study conception, design, and revision of the final manuscript. NS, AF, ET, and MG: data collection. ZM: statistical analysis. AF, ET, and ZM: interpretation of results. ET, NS, MG, ZM, and AF: drafting of the manuscript. All authors contributed to the article and approved the submitted version.

## Conflict of interest

The authors declare that the research was conducted in the absence of any commercial or financial relationships that could be construed as a potential conflict of interest.

## Publisher’s note

All claims expressed in this article are solely those of the authors and do not necessarily represent those of their affiliated organizations, or those of the publisher, the editors and the reviewers. Any product that may be evaluated in this article, or claim that may be made by its manufacturer, is not guaranteed or endorsed by the publisher.
